# Ventricular assist device implantation in grown-up congenital heart disease patients

**DOI:** 10.1186/1749-8090-8-S1-O65

**Published:** 2013-09-11

**Authors:** O Szárszoi, J Pirk, J Maly, M Urban, D Turek, H Riha, T Kotulak, Z Dorazilova, I Netuka

**Affiliations:** 1 Department of Cardiac Surgery, Institute for Clinical and Experimental Medicine, Prague, Czech Republic; 2 Department of Cardiac Anaesthesiology, Institute for Clinical and Experimental Medicine, Prague, Czech Republic; 3 Department of Cardiology, Institute for Clinical and Experimental Medicine, Prague, Czech Republic

## Introduction

Advances in palliation of congenital heart disease have resulted in improved survival to adulthood. Nevertheless many of these patients ultimately develop end-stage heart failure requiring heart transplantation. Implantation of ventricular assist device is life-saving option for such patients, allowing for bridge to transplant. In the present study we describe our experience with implantation of ventricular assist device Heart Mate II as bridge to transplant in adult patients with congenital heart disease.

## Methods

Of 104 patients who underwent ventricular assist device implantation at our institute from December 2007 to April 2013, 8 patients ranging in age from 14 to 40 years (6 male), in the end-stage of the heart failure were diagnosed to have adult congenital heart disease. We retrospectively reviewed the clinical data, operative and postoperative courses of these patients.

## Results

Anatomical diagnoses were transposition of the great arteries in 6 patients (5 patients have Mustard procedure as a child and one patient has congenitally corrected transposition of the great arteries), single-ventricle defect in 1 patient and coarctation of aorta in 1 patient.

Despite high risk heart surgery, implantation of ventricular assist device was performed in all patients with zero perioperative mortality. Overall mechanical support time was 232 ± 116 days (mean ± SD; range 34 - 349 days). Four patients were successfully bridged to heart transplantation, three patients unfortunately suffered from thromboembolic and septic complications and had died, and one is still waiting for heart transplant.

## Conclusion

Adults with congenital heart defects and congestive heart failure are a challenging population because of their complex anatomy, prior surgical palliation, and hemodynamic status. We conclude from our experience that ventricular assist device surgery can be safely performed in these high risk patients.

Study was supported by Grant IGA MZ NT/11269–5.

